# Maternal Hypertensive Pregnancy Disorders and Mental and Behavioral Disorders in the Offspring: a Review

**DOI:** 10.1007/s11906-021-01141-w

**Published:** 2021-05-13

**Authors:** Rachel Robinson, Anna Lähdepuro, Soile Tuovinen, Polina Girchenko, Ville Rantalainen, Kati Heinonen, Jari Lahti, Katri Räikkönen, Marius Lahti-Pulkkinen

**Affiliations:** 1 Department of Psychology and Logopedics, Faculty of Medicine, University of Helsinki, Haartmaninkatu 3, P.O. Box 9, FI-00014 University of Helsinki, Helsinki, Finland; 2 National Institute for Health and Welfare, Helsinki, Finland; 3 Queens Medical Research Unit, University of Edinburgh, Edinburgh, UK

**Keywords:** Preeclampsia, Hypertension, Mental disorders, Prenatal, Etiology, Psychopathology

## Abstract

**Purpose of Review:**

We review here recent original research and meta-analytic evidence on the associations of maternal hypertensive pregnancy disorders and mental and behavioral disorders in the offspring.

**Recent Findings:**

Seven meta-analyses and 11 of 16 original research studies published since 2015 showed significant associations between maternal hypertensive pregnancy disorders and offspring mental and behavioral disorders. Evidence was most consistent in meta-analyses and high-quality cohort studies. The associations, independent of familial confounding, were observed on different mental and behavioral disorders in childhood and schizophrenia in adulthood. Preterm birth and small-for-gestational age birth emerged as possible moderators and mediators of the associations. Cross-sectional and case-control studies yielded inconsistent findings, but had lower methodological quality.

**Summary:**

Accumulating evidence from methodologically sound studies shows that maternal hypertensive pregnancy disorders are associated with an increased risk of mental and behavioral disorders in the offspring in childhood. More studies on adult mental disorders are needed.

**Supplementary Information:**

The online version contains supplementary material available at 10.1007/s11906-021-01141-w.

## Introduction

Hypertensive pregnancy disorders, including chronic hypertension, gestational hypertension, preeclampsia, and eclampsia complicate up to 5–8% of all pregnancies [1]. Meta-analytic evidence shows that hypertensive pregnancy disorders predict an increased risk of cardiovascular disease and premature mortality in the mother [2–4] and of preterm birth [5, 6], small for gestational age (SGA) birth [5], stillbirth and neonatal death [5], and higher systolic and diastolic blood pressure and body mass index (BMI) [7] in the offspring.

Especially in recent years, an increasing amount of studies have also assessed the effects of maternal hypertensive pregnancy disorders on offspring mental and behavioral disorders [8–14]. In light of this and since earlier original research studies have been reviewed thoroughly in previous meta-analyses [15•, 16, 17–22], we reviewed the recent evidence from meta-analytic and new original research studies on maternal hypertensive pregnancy disorders and offspring mental and behavioral disorders published since 2015. We focus here on diagnosed mental and behavioral disorders, as classified in the International Classification of Diseases and Related Conditions, Tenth Revision (ICD-10) with diagnostic codes F00-F99, as outcomes [23].

## Methods

We searched Medline, Google Scholar, and Science Direct databases on original research and review articles with the search words “hypertensive pregnancy disorder” or “preeclampsia” or “gestational hypertension” and “mental disorder” or “psychiatric,” “schizophrenia” or “depression” or “bipolar” or “anxiety” or “autism” or “eating disorder” or “substance use disorder” or “ADHD” or “conduct disorder” or “personality disorder”. We also examined reference lists of the identified articles for additional references. We focused our search on articles published since 2015. The corresponding author went through the search results and excluded duplicates, narrative and systematic reviews, and other studies not providing any data on our study question. We also excluded studies focusing solely on the symptoms of mental and behavioral disorders.

Two authors (RR, MLP) conducted a quality of evidence assessment of the new original findings according to the Newcastle-Ottawa Scale (NOS) assessment criteria. The evaluated studies were cohort, cross-sectional, and case-control studies, each rated according to the criteria appropriate for the particular study type [24, 25]. The NOS scales for case-control and cohort studies yield a maximum of nine stars and the scale for cross-sectional studies a maximum of ten stars. A higher number of stars indicate higher methodological quality. In one cohort study, one evaluator, MLP, was an author, and hence to avoid bias, RR conducted this NOS assessment together with AL. In cases of disagreement in assessment, we reached consensus by discussion.

Supplementary Table 1, Supplementary Table 2, and Supplementary Table 3 in the Online Data Supplement provide our specific assessment criteria for the study questions at hand, which we predefined before the start of the assessment and systematically applied in duplicate to all studies. To sum, we assessed the statistical methods based on whether the study used sibling comparisons and whether the study accounted for familial confounding by maternal and/or paternal mental disorders, took into account cardiometabolic conditions of maternal prepregnancy overweight/obesity and/or diabetes disorders, and considered the mediating or moderating effects of preterm and/or SGA birth. Additional assessment criteria included whether maternal hypertensive pregnancy disorder and/or offspring mental and behavioral disorder diagnoses were physician-diagnosed from structured interviews, medical records, or health registers vs. retrospective self- or maternal self-reports of diagnosis. We assessed the representativeness of the exposed and selection of the non-exposed groups, attrition bias, and the adequacy of the length of follow-up for the child to develop the outcome in question.

## Results

### Meta-Analyses

Our literature search yielded seven meta-analyses on maternal hypertensive pregnancy disorders and offspring mental and behavioral disorders since 2015 [15•, 16, 17–21]. Table 1 summarizes their study designs, study questions, and key results. Five meta-analyses focused on autism spectrum disorders (ASD), two on attention-deficit hyperactivity disorder (ADHD), and one on schizophrenia. The five ASD meta-analyses included three to 21 studies with 8000 to 7.5 million participants [15•, 16, 18•, 19•, 21]. The two ADHD meta-analyses included 8 and 10 studies with at most over a million participants [15•, 20]. The preeclampsia and offspring schizophrenia meta-analysis included 11 studies with 1.4 million participants [17]. In all seven meta-analyses, maternal preeclampsia was associated with increased risks of the assessed neuropsychiatric disorders. Any maternal hypertensive pregnancy disorder and specifically gestational and/or chronic hypertension was associated with increased ASD risk in two and increased ADHD risk in one meta-analysis. All odds or risk ratios for the effects of different maternal hypertensive pregnancy disorders on offspring ASD, ADHD, and schizophrenia risk varied between 1.3- and 1.7-fold (95% confidence intervals (CIs) varying from 1.0 to 2.2).

**Table 1:** Meta-analyses on the associations of maternal hypertensive pregnancy disorders and mental and behavioral disorders in the offspring since 2015. Key study characteristics and results

Study	Study types	Number of studies	Sample size	Exposure	Diagnostic method for hypertensive pregnancy disorders	Offspring diagnostic outcome	Diagnostic method for offspring mental disorders	Covariates	Key results
Dachew et al. [16]	Cohort (*n*=4) and case-control (*n*=6)	10	1,166,307	Preeclampsia	Medical records, registries, or databases	ASD	ICD-9, ICD-10, DSM-III-R, DSM-IV, ADI-R	Seven studies: child sex. Five studies: maternal age and prenatal substance use. Other covariates: assessed seldom.	Maternal preeclampsia was associated with an increased risk of ASD in the offspring (RR=1.3, 95% CI=1.2–1.5). No marked heterogeneity in effect sizes.
Dachew et al. [17]	Cohort (*n*=4) and case-control (*n*=7)	11	1,462,527	Preeclampsia	Medical records and diagnostic assessments	Schizophrenia	ICD-8, ICD-9, ICD-10, DSM-IV	Maternal age and child sex, otherwise varying across studies.	Maternal preeclampsia was associated with an increased risk of schizophrenia (RR=1.4, 95% CI=1.1–1.7). The effect was present in cohort (RR=1.8, 95% CI=1.2–2.7) but not case-control studies (RR=1.2, 95% CI=0.9–1.6).
Maher et al. [15•]	Cohort, case-control and cross-sectional	20 studies for ASD and 10 studies for ADHD	ASD: 941,285 in unadjusted and 777,518 adjusted and analyses ADHD: 1,428,209 in unadjusted and 1,395,605 in adjusted analyses	Hypertensive disorders of pregnancy; preeclampsia and other hypertensive disorders of pregnancy	Medical records or self-reports of physician diagnosis	ASD and ADHD	Varying criteria: physician diagnosis, symptom completion criteria, maternal reports, or diagnostic interviews	Varying across studies.	Maternal hypertensive disorders of pregnancy predicted increased offspring risks of ASD (aOR=1.4, 95% CI=1.1–1.6) and ADHD (aOR=1.3, 95% CI=1.2–1.4), independently of covariates. Preeclampsia independently predicted increased risks of ASD (OR=1.4, 95% CI=1.1–1.8; aOR=1.5, 95% CI=1.3–1.8) and ADHD (OR=1.3, 95% CI=1.2–1.4; aOR=1.3, 95% CI=1.2–1.4). Other hypertensive disorders of pregnancy were associated with increased ASD risk (OR=1.4, 95% CI=1.2–1.7) but not in adjusted models (OR=1.3, 95% CI=0.9–1.7). They did independently predict increased ADHD risk (OR=1.6, 95% CI=1.1–2.5; aOR=1.7, 95% CI=1.1–2.7).
Jenabi et al. [18•]	Cohort (*n*=6) and case-control (*n*=7)	13	7,561,696	Preeclampsia	N/S	ASD	ICD-9, ICD-10, DSM-IV, DSM-5, ADI-R, ADOS	Maternal age, psychosocial disorders, parity, smoking, child sex, birth year, birth hospital and year of diagnosis, prenatal care	Maternal preeclampsia was associated with an increased risk of ASD in the offspring (RR from 6 studies=1.3, 95% CI=1.2–1.4; OR from 7 studies=1.4, 95% CI=1.1–1.6; unadjusted OR=1.5, 95% CI=0.8–2.2; adjusted OR=1.4, 9% CI=1.1–1.6)
Wang et al. [26]	Cohort (*n*=1) and case-control (*n*=2)	3	8118	Preeclampsia	N/S	ASD	ICD-9, ICD-10	Not Specified	Maternal preeclampsia predicted increased offspring risk of ASD (RR=1.5, 95% CI=1.0–2.2).
Xu et al. [19•]	Cohort and case-control	21; 11 on preeclampsia, 9 on gestational hypertension, 4 on chronic hypertension, 3 on mixed hypertensive pregnancy disorders	6,527,652	Hypertensive disorders of pregnancy; preeclampsia, gestational hypertension, chronic hypertension and mixed	N/S	ASD	DSM-III, DSM-III-R, DSM-IV, ICD-8, ICD-9, ICD-10, ADI-R, ADOS. In 3 studies, NS.	Stratified analyses by maternal education and age, preterm birth, premature rupture of membranes, geographic area, and child sex.	Maternal hypertensive disorders of pregnancy were associated with an increased risk of ASD (OR=1.4, 95% CI=1.3–1.5). Both preeclampsia ASD (OR=1.4, 95% CI=1.3–1.6), gestational hypertension ASD (OR=1.4, 95% CI=1.2–1.5), chronic hypertension ASD (OR=1.5, 95% CI=1.3–1.7) and mixed hypertension (OR=1.4, 95% CI=1.1–1.7) exposures were associated with increased risks of ASD.
Zhu et al. [20]	Cohort (*n*=1), case-control (*n*=7)	8	N/S	Preeclampsia	N/S	ADHD	Medical register or interview-based	Varying matching factors in different studies	Maternal preeclampsia was associated with an increased risk of ADHD in the offspring (OR=1.3, 95% CI=1.2–1.4).

ASD=autism spectrum disorder; ADHD=attention-deficit hyperactivity disorder; DSM=Diagnostic and Statistical Manual for Mental Disorders; ADI-R= Autism Diagnostic Interview-Revised; ADOS= Autism Diagnostic Observation Schedule; ICD=International Classification of Diseases and Related Conditions; OR=odds ratio; aOR=adjusted odds ratio; RR=risk ratio; aRR=adjusted risk ratio; CI=confidence interval; N/S=not specified

The meta-analyses also presented adjusted effect size estimates. Most often, the effects of maternal hypertensive pregnancy disorders were independent of any assessed covariates but in one meta-analysis, maternal preeclampsia but not chronic or gestational hypertension independently predicted increased offspring ASD risk [15•].

However, the meta-analyses could not comprehensively assess the roles played by different potential confounders, mediators, and/or moderators, as the covariates used varied across studies [15•, 16, 17–20]. Possible key confounding factors or moderators include familial confounding by maternal/parental mental health, other genetic or shared familial environmental influences, and maternal metabolic disorders during pregnancy (diabetes disorders and early pregnancy overweight/obesity). All these factors are highly comorbid with hypertensive pregnancy disorders [1, 11, 27–29] and predict increased offspring risk of mental and behavioral disorders [30–34]. Furthermore, hypertensive pregnancy disorders increase the risk of preterm and SGA birth [5]. Preterm and SGA birth predict an increased risk of mental disorders [35, 36], and they may mediate or moderate the effects of hypertensive pregnancy disorders on offspring mental and behavioral disorders [19•]. Discussed next, some of the recent original research studies examined these confounding factors, mediators, and moderators more thoroughly.

### Original Research Studies

Our literature search yielded 23 new peer-reviewed original research studies on the associations between maternal hypertensive pregnancy disorders and offspring mental disorders since 2015 (Table 2). Seven [46–52] of which were included in the meta-analyses described above and their individual study findings are not described in more detail to avoid duplicate emphasis on the same studies.

**Table 2:**
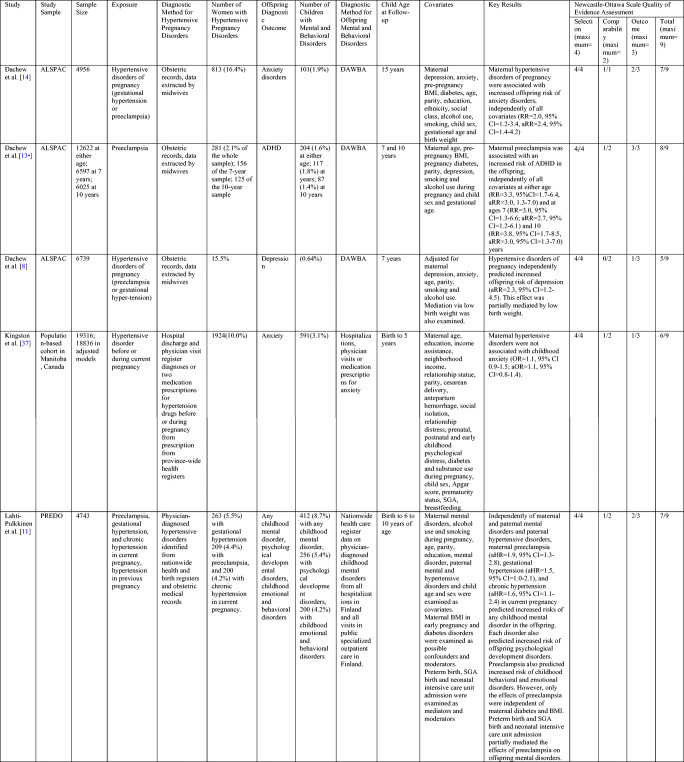

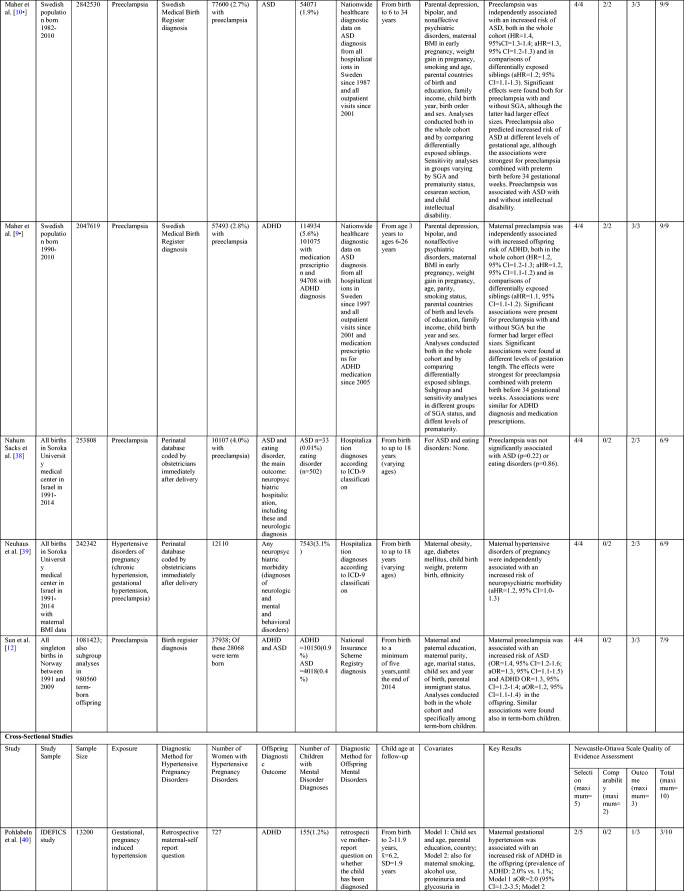

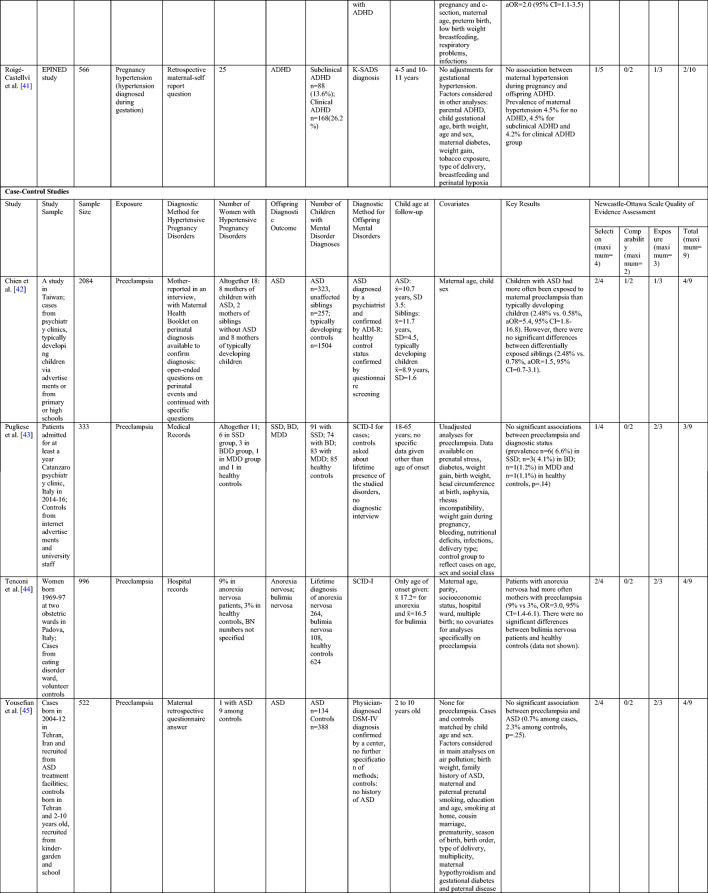
Study characteristics and quality of evidence assessment of the new original research studies on maternal hypertensive pregnancy disorders and offspring mental and behavioral disorders

*ADI-R* Autism Diagnostic Interview-Revised; *ADHD* attention deficit hyperactivity disorder; *ADOS* Autism Diagnostic Observation Schedule; *aHR* adjusted hazard ratio; *ALSPAC* Avon Longitudinal Study of Parents and Children; *aOR* adjusted odds ratio; *aRR* adjusted risk ratio; *ASD* autism spectrum disorder; *BD* bipolar disorder; *CI* Confidence Interval; *DAWBA* Development and Well-being Assessment; *DSM* Diagnostic and Statistical Manual for Mental Disorders; *MDD* major depressive disorder; *EPINED* Epidemiological Study of Neurodevelopmental Disorders; *HR* hazard ratio; *ICD* International Classification of Diseases and Related Conditions; *K-SADS* Kiddie Schedule for Affective Disorders and Schizophrenia *OR* odds ratio; *PREDO* Prediction and Prevention of Preeclampsia and Intrauterine Growth Restriction; *RR* risk ratio; *SD* standard deviation; *SCID* Structured Clinical Interview for DSM-IV; *SGA* small for gestational age; *SSD* schizophrenia spectrum disorder; *x̄* sample mean

The remaining 16 original research articles report data from 12 different study samples. Ten studies employed cohort and two were cross-sectional and four case-control study designs. Table 2 shows the study design, covariates, sample sizes, diagnostic methods, and results of the studies along with the summary of the NOS assessment of the quality of evidence in these studies. Supplementary Table 1, Supplementary Table 2, and Supplementary Table 3 in the online Data Supplement provide more information on these assessments. Supplementary Table 4 in the Online Data Supplement specifies the diagnostic criteria and diagnostic methods used for maternal hypertensive pregnancy disorders in the different studies.

#### Cohort Studies

Of the ten cohort studies [8–14, 37–39], eight reported significant associations between maternal hypertensive pregnancy disorders and increased offspring risk of mental and behavioral disorders and two reported null findings. All cohort studies had many methodological strengths and received 5–9 stars in the NOS assessment (Table 2 and Supplementary Table 1 in the Online Data Supplement). They all used a prospective study design and objective nationwide or statewide medical or obstetric register data on physician-diagnosed maternal hypertensive pregnancy disorders and diagnostic register or structured interview-based data on offspring mental and behavioral disorders.

Two publications from a Swedish population-wide cohort among over two million participants received the highest NOS rating [9, 10]. These studies showed that maternal preeclampsia predicted an increased, 1.1–1.2-fold (95% CIs=1.1–1.3) risk of ADHD [9•] and 1.2–1.4-fold (95% CIs=1.1–1.4) risk of ASD [10•] in the offspring. The findings also suggested that familial confounding did not explain the associations, since significant effects were observed in the whole population and in comparisons of differentially exposed siblings. Neither did parental mental disorders nor maternal early pregnancy BMI explain the associations [9, 10]. However, maternal diabetes was unaccounted for. In both the whole cohort and sibling comparisons, preeclampsia predicted ASD and ADHD when occurring together with or without SGA birth. The effects were stronger if the mother had preeclampsia and the child was born SGA. In the full cohort, preeclampsia was associated with offspring ASD and ADHD in term-born and preterm offspring. Additive effects of preeclampsia and preterm birth were also observed. However, as a limitation, these studies did not examine mediation or moderation by preterm birth in the sibling comparisons [9, 10], although pregnancies with preeclampsia more often lead to preterm births than pregnancies without preeclampsia [5, 6].

Three large studies conducted in the prospective Avon Longitudinal Study of Parents and Children (ALSPAC) cohort received eight, seven, and five stars in our NOS assessment [8, 13•, 14]. They each showed significant effects of maternal hypertensive pregnancy disorders on offspring mental and behavioral disorders. Two ALSPAC studies among 6739 and 5231 mother-child dyads, respectively, showed that maternal hypertensive pregnancy disorders, defined as either gestational hypertension or preeclampsia, predicted 2.3-fold (95% CI=1.2–4.5) risk of depression in 7-year-old children [8] and 2.4-fold (95% CI=1.2–3.4) risk of anxiety disorders in 15-year-old offspring [14]. The third study, with the highest methodological quality, showed among 12,000 participants that maternal preeclampsia predicted 2.7–3.8-fold (95% CIs=1.2–8.5) risk of ADHD in 7- and 10-year-old offspring [13•]. All three studies had representative study samples [8, 13•, 14]. The studies on anxiety and ADHD considered potential confounders carefully, and the effects of hypertensive pregnancy disorders or specifically preeclampsia were independent of maternal diabetes, depression, and BMI in pregnancy and child gestational age [13•, 14]. The effects on anxiety disorders were also independent of maternal prenatal anxiety and child birth weight [14]. The study on depression considered fewer covariates, but the effect of maternal preeclampsia or gestational hypertension on offspring depression was independent of maternal prenatal depressive and anxiety symptoms and partially mediated by low birth weight [8]. However, the generalizability of the findings of the depression and anxiety studies is limited by noticeable follow-up attrition [8, 14].

Three representative prospective cohort studies from Norway [12], Canada [37], and Finland [11] each rated as having good methodological quality received seven stars in the NOS assessment. The Norwegian study among over one million mother-child dyads showed 1.3–1.4-fold (95% CIs=1.1–1.6) increased risk of ASD and 1.2–1.3-fold (95% CIs=1.1–1.4) increased risk of ADHD in offspring exposed to maternal preeclampsia [12]. Preeclampsia showed similar effects in the whole cohort and among term-born offspring. While this study accounted for many sociodemographic factors, it did not control for parental mental disorders or maternal metabolic disorders [12]. Contrastingly, the Canadian study [37] of over 19,000 participants did control for parental mental health, maternal metabolic disorders, and child preterm and SGA birth. The study found no associations between maternal hypertensive disorders and offspring anxiety disorders in early childhood. As a limitation, the study authors did not specify whether maternal hypertensive disorders were present before or during the index pregnancy [37]. In comparison, in the Prediction and Prevention of Preeclampsia and Intrauterine Growth Restriction (PREDO) cohort, we showed among over 4700 participants that maternal chronic hypertension, gestational hypertension, and preeclampsia in the current pregnancy each predicted significantly increased 1.5–1.9 fold (95% CIs=1.0–2.8) risks of any childhood mental disorder and psychological development disorders in the offspring [11]. Preeclampsia also predicted an increased risk of childhood emotional and behavioral disorders. All associations were independent of maternal and paternal mental disorders and paternal hypertensive disorders. However, only the effects of maternal preeclampsia were independent of diabetes disorders and overweight/obesity in early pregnancy. Furthermore, preterm and SGA birth both partially mediated the effects of preeclampsia on offspring childhood mental disorders. No effects were found for maternal hypertensive disorders present before the current pregnancy, which included hypertensive pregnancy disorders diagnosed in previous pregnancies and chronic hypertension diagnosed only before the current pregnancy [11].

Two representative studies, each receiving six out of nine NOS stars, reported data from an Israeli cohort of over 240,000 participants [38, 39]. The first [38] showed that maternal preeclampsia was independently associated with an increased offspring risk of certain neurological disorders but not with the assessed mental and behavioral disorders—ASD and eating disorders. However, these two disorders had very low prevalence, limiting statistical power to reliably assess them [38]. The other Israeli study showed that maternal hypertensive pregnancy disorders predicted 1.2-fold (95% CI=1.0–1.3) increased risk of any neuropsychiatric disorder, defined as any mental, behavioral, or neurological disorder in the offspring [39]. These effects were independent of maternal obesity, diabetes, preterm birth, and birth weight. The specific effects of maternal hypertensive pregnancy disorders on offspring mental and behavioral disorders were, however, not reported [39]. These Israeli studies did not control for familial confounding by parental mental disorders [38, 39].

In general, the cohort studies since 2015 show a relatively consistent pattern of maternal hypertensive pregnancy disorders predicting increased offspring mental and behavioral disorders in childhood and adolescence. As methodological strengths, in addition to the objective physician-diagnosed data on the exposures (Table 2 and Supplementary Table 4 in the Online Data Supplement) and outcomes (Table 2), all studies had good representativeness, a longitudinal study design starting from the pregnancy period, and the controls and cases with hypertensive pregnancy disorders were recruited from the same populations. There were also methodological limitations in all studies, as discussed. Furthermore, the Israeli studies [38, 39], the ALSPAC study on depression [8], the PREDO study [11], and the Canadian study [37] each ended their follow-ups at ages when many children had possibly not yet received their diagnosis. Further studies with longer follow-ups are needed. No cohort studies reported findings on mental disorders in adulthood (Table 2). When considering the width of the available evidence base, it is of note that three cohorts reported two or three studies on different mental and behavioral disorders, meaning that many same individuals were included in multiple individual studies.

#### Case-Control and Cross-Sectional Studies

The findings of the studies using cross-sectional and case-control study designs on maternal hypertensive pregnancy disorders and offspring mental and behavioral disorders are mixed, and they all had several methodological limitations (Table 2). In the larger cross-sectional study among 13,200 participants, maternal gestational hypertension was associated with a two-fold (95% CIs=1.1–3.5) increased ADHD risk in children [40]. However, since this study used maternal retrospective reports to identify both child ADHD and maternal gestational hypertension, shared method and recall bias may have influenced the findings [41, 46, 53]. The other cross-sectional study found no effects of maternal hypertension diagnosed during pregnancy on offspring ADHD at 3–4 or 11–12 years of age among 566 participants [41]. Although child ADHD was diagnosed with diagnostic interviews, maternal hypertensive disorder diagnosis was based on maternal retrospective self-reports. Furthermore, neither cross-sectional study adequately controlled for key covariates or attrition effects. These and other methodological limitations resulted in grading these studies with only two [41] and three [40] out of possible ten NOS stars (Table 2 and Supplementary Table 2 in the Online Data Supplement).

Of the four case-control studies, two found no associations between maternal hypertensive pregnancy disorders and offspring mental and behavioral disorders, while two studies reported mixed findings (Table 2). An Italian study among 333 participants found no significant differences in the prevalence of maternal preeclampsia, identified from medical records, between adult offspring with physician-diagnosed schizophrenia, major depressive disorder, bipolar disorder, and healthy controls [43]. However, the low number of participants in each diagnostic group limits the reliability of the findings [43]. Two case-control studies [42, 45] on maternal preeclampsia and offspring ASD used possibly biased retrospective self-reports of maternal preeclampsia. Of these, a study in Iran among 522 participants found no effects of maternal preeclampsia on child ASD [45]. While in a Taiwanese study among 2084 participants, children with ASD more often had mothers with preeclampsia than typically developing children. However, the study showed no differences between siblings with and without ASD, suggesting familial confounding [42]. Nevertheless, both studies had high likelihoods of false positive and false negative findings due to the low number of women with preeclampsia. Finally, a study among approximately 1000 mothers and their female offspring showed a significant association between maternal preeclampsia and increased offspring risk of anorexia nervosa, but not bulimia nervosa [44]. This study used register and interview data for maternal and child diagnoses [44].

However, all four case-control studies had methodological limitations and received only three or four out of nine stars in the NOS assessment (Table 2 and Supplementary Table 3 in the Online Data Supplement). For example, while the case-control study using sibling comparisons adjusted their analyses for maternal age and child sex [42], the three other studies did not control for any covariates. Selection of cases and controls did not follow the same methods in any case-control study: controls were recruited from different communities than cases, and only one study certified mental disorder diagnosis with the same method for both cases and controls (Table 2). Also, two of the four case-control studies used maternal retrospective self-reports to diagnose maternal hypertensive pregnancy disorders, and none of them specified the diagnostic criteria used to classify these maternal conditions (Supplementary Table 4 in the Online Data Supplement). These factors limit the validity of the case-control study findings [42–45].

## Discussion

The findings of the recent meta-analyses and cohort studies consistently point to the predisposing effects of maternal hypertensive pregnancy disorders and especially preeclampsia on offspring mental and behavioral disorders in childhood. The expanding evidence base includes findings among altogether millions of participants. Findings from cross-sectional and case-control studies, in turn, are very inconsistent, but notably, the same studies have had important limitations in methodological quality.

Hence, several cohort studies and meta-analyses yield a coherent picture of replicated associations between maternal hypertensive pregnancy disorders and increased risk of mental and behavioral disorders in children. The same increasing body of evidence suggests that these effects of maternal hypertensive pregnancy disorders are independent of maternal overweight/obesity and diabetes disorders and familial confounding by maternal or paternal mental disorders. However, only the Swedish population-wide studies on ASD and ADHD and the case-control study in Iran on ASD assessed familial confounding more soundly via comparisons of differentially exposed siblings [9, 10, 42], and no sibling comparison data exists on other mental and behavioral disorders than ASD or ADHD. Furthermore, while preterm and SGA birth have emerged as possible moderators or partial mediators of the effects of hypertensive pregnancy disorders on offspring mental and behavioral disorders [8–11], mediation or moderation by preterm birth was not addressed in any of the sibling comparisons [9, 10, 42]. Although maternal hypertensive pregnancy disorders consistently predicted increased risks of ASD and ADHD, the effect sizes for these most commonly studied disorders were relatively small in the most representative studies. Maternal hypertensive pregnancy disorders thus constitute one of many risk factors for these neuropsychiatric disorders, with small but significant effect sizes. Interestingly, the authors of the Swedish cohort studies later showed that offspring risks of ASD and ADHD were even higher if both the grandmother and mother had had preeclampsia, suggesting multigenerational effects [54], and a novel avenue for research.

While there are numerous studies on mental and behavioral disorders in childhood and adolescence, and meta-analytic evidence of associations between maternal preeclampsia on offspring schizophrenia in adulthood, very few studies have examined the effects on other adulthood mental disorders. An early cohort study showed that maternal gestational hypertension but not preeclampsia predicted an increased risk of severe mental disorders in adult offspring [55]. Two case-control studies reviewed here had adulthood follow-ups, one on major depression, schizophrenia, and bipolar disorder [43] and the other on eating disorders [44]. These studies produced mixed findings in a restricted number of exposed individuals [43, 44]. Hence, no clear conclusions can be made of effects on other adult mental disorders. Also regarding child and adolescent mental disorders, the studies have either focused on ADHD, ASD, any mental disorder, psychological development disorders, childhood behavioral and emotional disorders, anxiety, and depression as outcomes. In contrast, our literature search yielded no studies specifically on conduct disorders, personality disorders, or substance use disorders. Thus further research needs to examine how widespread the effects of maternal hypertensive pregnancy disorders are on different mental and behavioral disorders, particularly on externalizing disorders.

An additional question of the effects of hypertensive pregnancy disorders on offspring mental and behavioral disorders is whether dose-response associations exist, i.e., the effects become more evident when the hypertensive pregnancy disorder is more severe. According to the ICD-10, preeclampsia can be classified according to its severity to mild/moderate and severe subtypes [23]. Some of the international guidelines for the treatment of hypertensive pregnancy disorders do not recommend the use of the severity classification in clinical practice as all preeclampsia cases can have dire consequences for the mother and her child [56]. However, the severity classification, dose-response effects, may provide important insights on potential causality. Three studies since 2015 assessed preeclampsia severity effects on offspring mental and behavioral disorders [11, 38, 46]. In PREDO, the more severe the maternal preeclampsia, the higher the offspring risk for childhood mental disorders [11]. Also, severe but not mild/moderate preeclampsia had effects that were independent of maternal early pregnancy BMI and diabetes disorders [11]. One study included in the meta-analyses on ASD defined severe preeclampsia as either a note of severe preeclampsia on a medical record, presence of HELLP syndrome, or preeclampsia combined with placental insufficiency [46]. This exposure was associated with strong effects on ASD and developmental delay [46]. In contrast, in the Israeli cohort study, preeclampsia severity was not associated with offspring ASD or eating disorders [38].

While maternal hypertensive pregnancy disorders have now repeatedly shown effects on offspring mental and behavioral disorders that are independent of maternal diabetes and/or prepregnancy obesity, only one study assessed additive effects of these three types of cardiometabolic conditions [11]. In that study, maternal hypertensive pregnancy disorders, diabetes disorders, and overweight/obesity in current pregnancy additively increased the risk of mental and behavioral disorders in children. While the cumulative incidence of childhood mental disorders was 7% among offspring of women with no maternal adverse cardiometabolic conditions in pregnancy, it was over 22% among offspring of women with all of these conditions [11]. Further studies are needed to replicate these findings.

The evidence of preterm birth, SGA birth, and low birth weight partially mediating the effects of preeclampsia on offspring mental and behavioral disorders [8, 11] suggests partially shared biological pathways underlying the effects of these conditions and maternal hypertensive pregnancy disorders on offspring mental health. Preeclampsia is a placental disorder characterized by placental insufficiency and SGA is often used in research as a proxy for placental insufficiency [9, 10, 46]. Placental insufficiency and structural changes are associated with offspring psychopathology risk [46, 57] and these placental modifications may be among the biological pathways leading from hypertensive pregnancy disorders, particularly preeclampsia, to offspring psychopathology risk. Furthermore, preterm birth predicts an increased risk of mental and behavioral disorders [11, 35], possibly via structural and functional alterations in brain development [58, 59]. Such neurodevelopmental alterations may contribute to the effects of maternal hypertensive pregnancy disorders on offspring mental and behavioral disorders [60].

Maternal hypertensive pregnancy disorders may also increase the risk of offspring mental disorders via maternal and offspring changes in the inflammatory system and hypothalamus-pituitary-adrenal axis functioning. Such changes have been shown as a consequence of maternal hypertensive pregnancy disorders and in offspring with mental disorders [60–63]. On a molecular level, there may be pleiotropic genetic effects between maternal hypertensive pregnancy disorders and offspring mental and behavioral disorders and epigenetic changes may mediate these associations. The genetic risk factors for mental disorders and hypertension partially overlap [64] and epigenetic DNA methylation and gene expression changes are seen in offspring of women with hypertensive pregnancy disorders [65] and patients with mental disorders [62].

The findings reviewed here suggest a possible independent role for maternal hypertensive pregnancy disorders in the etiology of offspring mental and behavioral disorders. Considering the marked effects maternal hypertensive pregnancy disorders also have on maternal and offspring cardiovascular and neonatal morbidity and mortality, the public health impact of these common conditions is marked and widespread. Together, these findings indicate that the pharmaceutical and lifestyle interventions that have either proven effective or show promise on the treatment of maternal hypertensive pregnancy disorders [56] also may have buffering effects on the somatic and mental health of the mother and her offspring.

The limitations of the available evidence include the case-control and cross-sectional studies not fulfilling most criteria to ensure unbiased reporting related to the definitions of exposures and outcomes, comparability of selection of cases and controls, and controlling for key covariates. In contrast to the cohort studies, which classified hypertensive pregnancy disorders according to standardized international diagnostic guidelines, the case-control and cross-sectional studies most often used retrospective maternal self-report questionnaires and did not specify the diagnostic criteria they used for hypertensive disorders (Table 2 and Supplementary Table 4 in the Online Data Supplement). The retrospective self-reports are prone to bias, which may limit the validity of the diagnostic categories and the generalizability of the findings of these studies. However, in the current review, the method of exposure assessment was accounted for in the NOS Quality of Evidence assessment (Table 2 and Supplementary Table 1, Supplementary Table 2, and Supplementary Table 3 in the Online Data Supplement). Furthermore, the large-scale studies on maternal hypertensive pregnancy disorders and offspring mental and behavioral disorders have been conducted in relatively affluent societies, and it remains uncertain how generalizable the findings are to different populations with varying healthcare coverage and guidelines [66]. The guidelines for the treatment of hypertensive pregnancy disorders vary across countries [66], and how this affects the prognosis of offspring born from pregnancies complicated by hypertensive pregnancy disorders remains unknown. For example, the US treatment guidelines suggest induced delivery after 34 gestational weeks in pregnancies complicated by preeclampsia, while there is no such recommendation in Europe [1]. Studies on the similarities and differences of the associations of hypertensive pregnancy disorders with offspring mental and behavioral disorders in different countries are needed.

Furthermore, ethical reasons prohibit randomized controlled trials on the effects of maternal hypertensive pregnancy disorders on offspring mental and behavioral disorders. It is important to note that causality cannot be directly inferred from the epidemiological studies reviewed here. The prospective cohort studies nevertheless yield preliminary answers about the direction of associations. Future studies may approximate a causal design by examining in randomized clinical trials whether interventions that have proven effective for maternal hypertensive pregnancy disorders also prevent mental and behavioral disorders in the offspring. It also remains unknown whether the effects of maternal hypertensive pregnancy disorders on offspring mental and behavioral disorders are modified by familial risk for psychopathology. To our knowledge, no studies have examined interaction effects of polygenic risk scores or parental mental disorders with maternal hypertensive pregnancy disorders on offspring mental disorders. Studies using sibling comparisons while simultaneously taking into account all key confounders, mediators, and moderators will shed important new light on possible familial confounding. Furthermore, while two meta-analyses [15•, 19•] and one original research study [11] examined the specific effects of maternal gestational hypertension, chronic hypertension, and preeclampsia on offspring mental and behavioral disorders, most of the new research studies have focused either on preeclampsia as a sole exposure or on the combined effects of gestational hypertension and preeclampsia on offspring mental and behavioral disorders. More research is needed on the roles played by other maternal hypertensive pregnancy disorders. Finally, further studies should examine more thoroughly the effects of maternal hypertensive pregnancy disorders on offspring mental and behavioral disorders in adulthood and externalizing disorders at any age.

## Conclusions

A large amount of recent research has focused on the associations of maternal hypertensive pregnancy disorders on offspring mental disorders. The evidence from cohort studies and meta-analyses is increasingly consistent in suggesting that maternal hypertensive pregnancy disorders are associated with increased risks of a wide range of different mental and behavioral disorders in childhood and adolescence, and schizophrenia in adulthood. Particularly consistent and convincing evidence exists on ASD and ADHD. While similar findings have been observed on other offspring mental and behavioral disorders especially in childhood, these findings warrant replication. Furthermore, studies on externalizing disorders and common adult mental disorders are scarce. Compared to other maternal hypertensive pregnancy disorders, the evidence is most consistent for maternal preeclampsia as a risk factor for offspring mental and behavioral disorders, and the available evidence suggests that the effects are independent of familial confounding. Maternal hypertensive pregnancy disorders are associated with an increased risk of mental and behavioral disorders in the offspring.

## Supplementary information

ESM 1 (PDF 201 kb)
