# Supplementary material for: Maternal Hypertensive Pregnancy Disorders and Mental and Behavioral Disorders in the Offspring: a Review
Source: Curr Hypertens Rep. 2021 May 13;23(5):30. doi: 10.1007/s11906-021-01141-w (PMC8116290; doi:10.1007/s11906-021-01141-w)
Supplement: Supplementary file 1 — (PDF 201 kb) [file 11906_2021_1141_MOESM1_ESM.pdf]

Rachel Robinson, Anna Lähdepuro, Soile Tuovinen, Polina Girchenko, Ville Rantalainen, Kati Heinonen, Jari Lahti, Katri Räikkönen, Marius Lahti-Pulkkinen. Maternal Hypertensive Pregnancy disorders and Mental and Behavioral disorders in the offspring: A Review. Manuscript Submitted to Current Hypertension Reports. Online Data Supplement.

**Supplementary Table 1.** Newcastle–Ottawa Scale for assessment of quality of cohort studies (If the acceptable criteria per domain was fulfilled then the study received a star.)											
Quality assessment criteria	Acceptable Criteria (★)	Dachew et al., 2019a(1)	Dachew et al., 2019b(2)	Dachew et al., 2020(3)	Kingston et al., 2015(4)	Lahti-Pulkkinen et al., 2020(5)	Maher et al., 2020a(6)	Maher et al., 2020b(7)	Nahum Sacks et al., 2019(8)	Neuhaus et al., 2020(9)	Sun et al., 2020(10)
**Selection**	★★★★	★★★★	★★★★	★★★★	★★★★	★★★★	★★★★	★★★★	★★★★	★★★★	★★★★
Representativeness of the exposed cohort	Representative of all women with hypertensive pregnancy disorders. Not specifically recruited based on a single risk factor for hypertensive pregnancy disorders, sample characteristics proportionate of the general population, no extreme disproportion-minimum 100 participants in each exposure and comparison group.	★	★	★	★	★	★	★	★	★	★
Selection of the non-exposed cohort	Drawn from same community as exposed cohort.	★	★	★	★	★	★	★	★	★	★
Ascertainment of exposure	Diagnoses of maternal hypertensive disorders from medical records or birth registers. Not based on maternal or offspring report.	★	★	★	★	★	★	★	★	★	★
Demonstration that outcome of interest was not present at start of study	Longitudinal follow-up study starting from birth. Outcome could not have occured before birth.	★	★	★	★	★	★	★	★	★	★
**Comparability**	★★	★	★	-	★	★	★★	★★	-	-	-
Comparability of cohorts on the basis of the design or analysis	Study controls for genetic confounding (using a sibling comparison design/control for polygenic risk for hypertension or mental disorders).	-	-	-	-	-	★	★	-	-	-
Study controls for or else takes into account at least 3 additional risk factors	Study controls for (or otherwise takes statistically into account) maternal or paternal mental health and maternal diabetes or obesity and preterm birth, small for gestational age birth or birth weight.	★	★	-	★	★	★	★	-	-	-
**Outcome**	★★★	★★	★★★	★	★★	★★	★★★	★★★	★★	★★	★★★
Assessment of outcome	Diagnostic interview or medical register diagnosis of mental disorders.	★	★	★	★	★	★	★	★	★	★
Was follow-up long enough for outcomes to occur	The follow-up extends to at least 6-7 yrs of age for ASD, 7-9 yrs for ADHD, 9 yrs for any childhood mental disorder, 11 yrs for anxiety disorders, 14 yrs for any mental and behavioral disorder, 18 yrs for eating disorders, 23 yrs for schizophrenia and 25-32 yrs for mood disorders.	★	★	-	-	-	★	★	-	-	★
Adequacy of follow up of cohorts	Complete follow-up, or subjects lost to follow-up unlikely to introduce bias (>50% follow up).	-	★	-	★	★	★	★	★	★	★
**Overall quality score (maximum=9)**		7	8	5	7	7	9	9	6	6	7

ASD=autism spectrum disorder; ADHD==attention deficit hyperactivity disorder; yrs=years.

In the study by Lahti-Pulkkinen et al. , 2020(5), a subsample (22%) was recruited based on the mother having one or more of risk factors for preeclampsia or intrauterine growth restriction. Other participants were recruited from antenatal clinic visits independently of their preeclampsia or intrauterine growth restriction status.

In the studies by Maher et al. 2020a, 2020b(6,7) and Sun et al., 2020(10), the follow-ups lasted for different lengths of time for different children. However, a large proportion of the cohorts did have adequate

**Supplementary Table 2.** Newcastle–Ottawa Scale assessment of the quality of the cross-sectional studies (each asterisk represents if individual criterion within the subsection was fulfilled).			
Quality assessment criteria	Acceptable(★)	Pohlabeln et al., 2017(11)	Roigé-Castellví et al., 2020(12)
**Selection**	★★★★★	★★	★
Representativeness of the sample	Representative of average pregnant women, (age/being at risk of disease, generalizability, random or non-random sampling)	★	★
Sample size	Justified and satisfactory, at least 100 per exposure group	★	-
Non-respondents	50% participation rate, participants and non-participants do not differ in prevalence of hypertensive pregnancy disorders or offspring mental disorders	-	-
Ascertainment of the Exposure	Diagnosis of hypertensive pregnancy disorders from obstetric records or medical records or registers★★	-	-
**Comparability**	★★	-	-
Comparability of cohorts on the basis of the design or analysis	Study uses sibling comparisons or accounts for polygenic risk factors for hypertension or mental disorders	-	-
Study controls for 3 additional risk factors	Study controls for (or otherwise takes into account) maternal or paternal mental health, maternal diabetes or overweight/obesity, and preterm birth, small for gestational age birth or birth weight	-	-
**Outcome**	★★★	★	★
Assessment of the Method	Structured clinical interview diagnosis★★<br>Health record diagnosis★	-	★
Statistical Test	The statistical assessment is clearly described, provides measurement error, confidence interval and probability level	★	-
**Overall quality score (maximum= 10)**		3	2

In the study by Roigé-Castellví et al., 2020(12), the cases and controls had different assessment methods for the assessment of child attention deficit and hyperactivity disorder.

Supplementary Table 3. Newcastle–Ottawa Scale assessment of the quality of case-control studies (If the acceptable criteria per domain was fulfilled then the study received a star).

Quality assessment criteria	Acceptable Criteria (★)	Chien et al., 2019(13)	Pugliese et al., 2019(14)	Tenconi et al., 2015(15)	Yousefian et al., 2018(16)
**Selection**	★★★★	★★	★	★★	★★
Is the Case Definition Adequate?	Physician-diagnosed mental fisorders from structured clinical interviews or health registers	★	★	★	★
Representativeness of the Cases	Representative of average patients with mental disorders, group size at least 100	★	-	-	★
Selection of Controls	Drawn from the same community as cases	-	-	-	-
Definition of Controls	No mental disorder diagnosis, status ascertained with same method as cases	-	-	★	-
**Comparability**	★★	★	-	-	-
Comparability of Cases and Controls on the Basis of the Design or Analysis	Study uses comparisons of differentially exposed siblings comparisons or accounts for polygenic risk scores for hypertension or mental disorders	★	-	`-	-
Study controls for at least 3 additional risk factors?	Study controls for (or otherwise takes statistically into account) maternal or paternal mental disorders, maternal diabetes or obesity and preterm birth, small for gestational age birth or birth weight	-	-	-	-
**Exposure**	★★★	★	★★	★★	★★
Ascertainment of Exposure	Diagnosis of hypertensive pregnancy disorders from medical registers or obstetric records	-	★	★	-
Same method of ascertainment for cases and controls (yes/no)	Same ascertainment method for both groups	★	★	★	★
Non-Response Rate	Less than 50% are non-respondents	-	-	-	★
**Overall quality score (maximum=9)**		4	3	4	4

The studies by Pugliese et al., 2019 (14)and Yousefian et al. 2018(16) did consider the three additional risk factors for other exposures but did not report any adjusted effect size estimates for the effects of preeclampsia on offspring mental and behavioral disorders.

Supplementary Table 4. Diagnostic criteria and definitions for hypertensive pregnancy disorders in the new research cohort, case-control and cross-sectional studies.		
**Study**	**Exposure**	**Diagnostic Methods and Criteria for and Diagnostic Definition of Hypertensive Pregnancy Disorders**
Dachew et al., 2019a(1)	Hypertensive disorders of pregnancy (gestational hypertension or preeclampsia)	Diagnoses were identified based on physician- or midwife measurements of blood pressure and proteinurea. Data on these measurements was extracted from obstetric records by trained midwives. The study used the International Society for the Study of Hypertension criteria to diagnose hypertensive disorders of pregnancy. All women were categorized into mutually exclusive categories of women with no hypertensive disorders of pregnancy or women with hypertensive disorders of pregnancy. Hypertensive disorders of pregnancy included gestational hypertension and preeclampsia. Preeclampsia was defined as systolic blood pressure ≥140 mmHg and/or diastolic blood pressure ≥90 mmHg, measured on ≥2 occasions after 20 weeks of gestation, with proteinuria(≥1 + on urine dipstick testing occurring at the same time as the elevated blood pressure), in a mother who did not report having hypertension prior to pregnancy. Gestational hypertension was defined with the same criteria of elevated blood pressure but without proteinuria.
Dachew et al., 2019b(2)	Preeclampsia	Diagnoses were identified based on physician- or midwife measurements of blood pressure and proteinurea. Data on these measurements was extracted from obstetric records by trained midwives. The study used International Society for the Study of Hypertension criteria. Women were classified into women with or without preeclampsia. Preeclampsia was defined as a systolic blood pressure (SBP) ≥140 mmHg or a diastolic blood pressure (DBP) ≥90 mmHg, measured on at least two occasions with proteinuria after 20 weeks of gestation, in mothers free of hypertension prior to pregnancy.
Dachew et al., 2020(3)	Hypertensive disorders of pregnancy (preeclampsia or gestational hyper-tension)	Diagnoses were identified based on physician- or midwife measurements of blood pressure and proteinurea. Data on these measurements was extracted from obstetric records by trained midwives. The study used International Society for the Study of Hypertension criteria. All women were categorized into mutually exclusive categories of women with or without hypertensive disorders of pregnancy. Hypertensive disorders of pregnancy included gestational hypertension and preeclampsia. Preeclampsia was defined as systolic blood pressure ≥140 mmHg and/or diastolic blood pressure ≥90 mmHg, measured on ≥2 occasions after 20 weeks of gestation, with proteinuria(≥1 + on urine dipstick testing occurring at the same time as the elevated blood pressure), in a mother who did not report having hypertension prior to pregnancy. Gestational hypertension was defined with the same criteria of elevated blood pressure but without proteinuria.
Kingston et al., 2015(4)	Hypertensive disorder before or during current pregnancy	Physician-diagnoses of hypertensive disorders before or during current pregnancy and prescriptions for hypertensive drugs were identified from province-wide health registers. These registers include data on hospital discharge and physician visits and medication purchases. The study grouped the participating women into two categories: women with and without hypertension. Hypertension was classified as present in women who had at least physician visit or hospitalization related to hypertension before pregnancy (ICD-9-CM codes 401–405 OR ICD-10-CA codes I10-I13, I15) or during pregnancy (ICD-9-CM code 642 OR ICD-10-CA codes 010-O16) , or two or more prescriptions for hypertension drugs.

Lahti-Pulkkinen et al., 2020(5)	Preeclampsia, gestational hypertension, and chronic hypertension in current pregnancy, hypertension in previous pregnancy	Physician-diagnoses of hypertensive pregnancy disorders were identified from the Finnish Medical Birth Register and Care Register for Health Care (HILMO) and from obstetric medical records. These registers contain physician-diagnosis from all hospital visits and specialized outpatient care visits in Finland. Diagnoses were coded into the registers according to the Finnish versions of the ICD. ICD-9 was in use in 1987-1995 and ICD-10 has been in use since 1996. Hypertensive pregnancy disorders were classified into separate subcategories, using the following criteria and diagnostic codes.<br>Chronic hypertension in current Pregnancy: ICD-10:O10, I10; Blood pressure ≥140/90 mmHg SBP/DBP present from pre-pregnancy or before 20 gestational weeks onwards<br>Gestational hypertension in current pregnancy: ICD-10:O13, Newly elevated blood pressure (≥140/90mmHg SBP/DBP) at ≥20 gestational weeks without proteinuria.<br>Preeclampsia in current pregnancy: ICD-10:O11,O14, O15; Newly elevated blood pressure (≥140 mmHg SBP and/or ≥90 mmHg DBP) at ≥20 gestational weeks combined with proteinuria (urinary excretion of ≥0.3 g protein in a 24-hour specimen or at least ++ in one or more or + dipstick in two consecutive measurements).<br>Unspecified hypertension in current pregnancy: ICD-10:O16, Transient hypertension during current pregnancy.<br>Hypertension only before current pregnancy: ICD-9: 642,401-405; ICD-10: I1, O10-O11, O13-O16; Has fulfilled the criteria for any of the specific disorders listed above, but only before the current pregnancy.
Maher et al., 2020a(6)	Preeclampsia	Physician-diagnoses of preeclampsia were identified from the Swedish Medical Birth Register. Medical doctors enter the diagnoses into the register, by reviewing discharge records and noting a diagnosis of preeclampsia at the time of discharge from the hospital using a standard form, containing the definition of preeclampsia, accompanied by an ICD-code and checkbox. Preeclampsia was defined as gestational hypertension (blood pressure ≥140/90 mmHg on/after 20 weeks' gestation) accompanied by proteinuria (≥0.3 g/day or ≥1 on a urine dipstick) or oedema. The following diagnostic codes of the Swedish ICD were used to identify preeclampsia: ICD-8 code 637: ICD-9 code 642 and ICD-10 code O14 or O15. ICD-8 was in use in Sweden until 1986, ICD-9 in 1987–1996 and ICD-10 has been in use from 1997 onwards.
Maher et al., 2020b(7)	Preeclampsia	Physician-diagnoses of preeclampsia were identified from the Swedish Medical Birth Register. Preeclampsia was diagnosed with the following criteria: Blood pressure ≥140/90 mmHg on or after 20 weeks' gestation combined with proteinuria (≥0.3 g/day or ≥1 on a urine dipstick on at least two occasions). Preeclampsia was identified with the following diagnostic codes of the Swedish versions of the ICD-9 and ICD-10: ICD-9 codes 642E-G and ICD-10 codes O14-O15.
Nahum Sacks et al., 2019(8)	Preeclampsia	Physician-diagnosis of preeclampsia. Diagnoses of preeclampsia were identified from a perinatal database where obstetricians coded the diagnoses immediately after delivery. Diagnostic criteria are not specified in text.
Neuhaus et al., 2020(9)	Hypertensive disorders of pregnancy (chronic hypertension, gestational hypertension, preeclampsia)	Physician-diagnosis of hypertensive disorders of pregnancy. Diagnoses of hypertensive disorders of pregnancy (chronic hypertension, gestational hypertension, preeclampsia) were identified from a perinatal database, where the diagnosed had been coded by obstetricians immediately after delivery. Diagnostic criteria were not specified in text.
Sun et al., 2020(10)	Preeclampsia	Physician-diagnosis of preeclampsia were identified from the Norwegian Medical Birth Registry. Preeclampsia was defined as blood pressure higher than 140/90 mmHg, an increase in systolic blood pressure ≥30 mm Hg, or an increase in diastolic blood pressure ≥15mm Hg and proteinuria (defined as a finding of at least +1 on dipstick urinalysis or at least 0.3g of protein in 24 hours), and/or edema after gestational age 20 weeks. Until 1998, preeclampsia was in Norway recorded to the birth register in a free-text field. Since1999, it has been reported by the selection of check boxes for mild preeclampsia, preeclampsia, preeclampsia before 34 weeks' gestation, eclampsia, and HELLP syndrome.

Pohlabeln et al., 2017(11)	Gestational, pregnancy induced hypertension	Diagnoses were based on maternal—retrospective self-report question answers. The women were asked whether they had been diagnosed with the following diagnosis during pregnancy: gestational hypertension (defined as having a blood pressure higher than 140/90 measured on two separate occasions, more than 6 h apart) or proteinuria.
Roigé-Castellví et al., 2020(12)	Pregnancy hypertension (hypertension diagnosed during gestation)	Diagnoses were based on maternal retrospective self-report questionnaires. The retrospective self-report questionnaire asked from the mothers whether or not they been diagnosed with hypertension during pregnancy. No diagnostic criteria were given.
Chien et al., 2019(13)	Preeclampsia	Mothers retrospectively self-reported the diagnosis in an interview. The interview included open-ended questions on perinatal events and continued with specific questions. The study also used Maternal Health Booklet on perinatal diagnosis to confirm the diagnosis. Preeclampsia was defined as blood pressure higher than 140/90 mmHg, proteinuria, and/or edema after gestational age 20 weeks.
Pugliese et al., 2019(14)	Preeclampsia	Diagnosis of preeclampsia were identified from medical records. Diagnostic criteria were not specified in text.
Tenconi et al., 2015(15)	Preeclampsia	Diagnosis of preeclampsia were identified from hospital records. Diagnostic criteria were not specified in text.
Yousefian et al., 2018(16)	Preeclampsia	Diagnoses were based on maternal retrospective self-report questionnaire answers. The diagnostic criteria or interview questions were not specified in the text.

REFERENCES

1. Dachew BA, Scott JG, Mamun A, Alati R. Hypertensive disorders of pregnancy and the risk of anxiety disorders in adolescence: Findings from the Avon Longitudinal Study of Parents and Children. J Psychiatr Res. 2019;110:159–65.
2. Dachew BA, Scott JG, Mamun A, Alati R. Pre-eclampsia and the risk of attention-deficit/hyperactivity disorder in offspring: Findings from the ALSPAC birth cohort study. Psychiatry Res. 2019;272:392–7.
3. Dachew BA, Scott JG, Betts K, Mamun A, Alati R. Hypertensive disorders of pregnancy and the risk of offspring depression in childhood: Findings from the Avon Longitudinal Study of Parents and Children. Dev Psychopathol. 2020;32(3):845–51.
4. Kingston D, Heaman M, Brownell M, Ekuma O. Predictors of childhood anxiety: A population-based cohort study. PLoS One. 2015;10(7): e0129339.
5. Lahti-Pulkkinen M, Girchenko P, Tuovinen S, Sammallahti S, Reynolds RM, Lahti J, et al. Maternal Hypertensive Pregnancy Disorders and Mental Disorders in Children. Hypertension. 2020;75(6):1429–38.
6. Maher GM, O'Keeffe GW, Dalman C, Kearney PM, McCarthy FP, Kenny LC, et al. Association between preeclampsia and autism spectrum disorder: a population-based study. J Child Psychol Psychiatry Allied Discip. 2020;61(2):131–9.
7. Maher GM, Dalman C, O'Keeffe GW, Kearney PM, McCarthy FP, Kenny LC, et al. Association between preeclampsia and attention-deficit hyperactivity disorder: a population-based and sibling-matched cohort study. Acta Psychiatr Scand. 2020;142(4):275–83.
8. Nahum Sacks K, Friger M, Shoham-Vardi I, Sergienko R, Spiegel E, Landau D, et al. Long-term neuropsychiatric morbidity in children exposed prenatally to preeclampsia. Early Hum Dev. 2019;130:96–100.
9. Neuhaus ZF, Gutvirtz G, Pariente G, Wainstock T, Landau D, Sheiner E. Maternal obesity and long-term neuropsychiatric morbidity of the offspring. Arch Gynecol Obstet. 2020;301(1):143–9.
10. Sun BZ, Moster D, Harmon QE, Wilcox AJ. Association of Preeclampsia in Term Births with Neurodevelopmental Disorders in Offspring. JAMA Psychiatry. 2020;77(8):823–9.
11. Pohlabeln H, Rach S, De Henauw S, Eiben G, Gwozdz W, Hadjigeorgiou C, et al. Further evidence for the role of pregnancy-induced hypertension and other early life influences in the development of ADHD: results from the IDEFICS study. Eur Child Adolesc Psychiatry. 2017;26(8):957–67.
12. Roigé-Castellví J, Morales-Hidalgo P, Voltas N, Hernández-Martínez C, van Ginkel G, Canals J. Prenatal and perinatal factors associated with ADHD risk in schoolchildren: EPINED epidemiological study. Eur Child Adolesc Psychiatry [Internet]. 2020;(0123456789). Available from: https://doi.org/10.1007/s00787-020-01519-2
13. Chien YL, Chou MC, Chou WJ, Wu YY, Tsai WC, Chiu YN, et al. Prenatal and perinatal risk factors and the clinical implications on autism spectrum disorder. Autism. 2019;23(3):783–91.
14. Pugliese V, Bruni A, Carbone EA, Calabrò G, Cerminara G, Sampogna G, et al. Maternal stress, prenatal medical illnesses and obstetric complications: Risk factors for schizophrenia spectrum disorder, bipolar disorder and major depressive disorder. Psychiatry Res. 2019;271:23–30.
15. Tenconi E, Santonastaso P, Monaco F, Favaro A. Obstetric complications and eating disorders: A replication study. Int J Eat Disord. 2015;48(4):424–30.
16. Yousefian F, Mahvi AH, Yunesian M, Hassanvand MS, Kashani H, Amini H. Long-term exposure to ambient air pollution and autism spectrum disorder in children: A case-control study in Tehran, Iran. Sci Total Environ. 2018;643:1216–22.
